# Hierarchical Lateral Control Scheme for Autonomous Vehicle with Uneven Time Delays Induced by Vision Sensors

**DOI:** 10.3390/s18082544

**Published:** 2018-08-03

**Authors:** Qi Liu, Yahui Liu, Congzhi Liu, Baiming Chen, Wenhao Zhang, Liang Li, Xuewu Ji

**Affiliations:** State Key Laboratory of Automotive Safety and Energy, Tsinghua University, Beijing 100084, China; q-liu16@mails.tsinghua.edu.cn (Q.L.); lcz17@mails.tsinghua.edu.cn (C.L.); cbm17@mails.tsinghua.edu.cn (B.C.); zhangwh17@mails.tsinghua.edu.cn (W.Z.); liangl@tsinghua.edu.cn (L.L.)

**Keywords:** autonomous driving, hierarchical controller, robust control, linear quadratic regulator (LQR), lateral tracking control, vision sensors, uneven sensor delays

## Abstract

Vision-based sensors are widely used in lateral control of autonomous vehicles, but the large computational cost of the visual algorithms often induces uneven time delays. In this paper, a hierarchical vision-based lateral control scheme is proposed, where the upper controller is designed by robust H_∞_-based linear quadratic regulator (LQR) algorithm to compensate sensor-induced delays, and the lower controller is based on logic threshold method, in order to achieve strong convergence of the steering angle. Firstly, the vehicle lateral model is built, and the nonlinear uncertainties induced by time delays are linearized with Taylor expansion. Secondly, the state space of the system is augmented to describe such uncertainties with polytopic inclusions, which is controlled by an H_∞_-based LQR controller with a low cost of online computation. Then, a lower controller is designed for the control of the steering motor. According to the results of the vehicle experiment as well as the hardware-in-the-loop (HIL) experiment, the proposed control scheme shows good performance in vehicle’s lateral control task, and exhibits better robustness compared with a conventional LQR controller. The proposed control scheme provides a feasible solution for the lateral control of autonomous driving.

## 1. Introduction

Autonomous driving is an effective way to reduce traffic accidents and to enhance driving experience. The basic purpose of the autonomous vehicle is to automatically drive the vehicle along specific trajectories without the driver’s intervention [1]. Taking into account a simple case, lane tracking, the task can be roughly summarized as making the vehicle follow the lane centerline or any other planned path by determining and performing the desired input [1,2], and this is required by all of levels of autonomous vehicles according to the outline of SAE-J3016 [3].

Assuming that all localization information is available, the path tracking becomes a motion control problem of the vehicle, which mainly includes lateral control and longitudinal control [2]. The lateral control aims to track a planned trajectory [4] through steer-by-wire (SBW) system or differential braking system [5,6,7,8,9], while the longitudinal control is to achieve closed-loop velocity control through drive-by-wire (DBW) and brake-by-wire system (BBW) [10]. Considering that longitudinal control already has mature commercial applications, such as cruise control (CC) and adaptive cruise control (ACC) [10,11], this study will mainly focus on the lateral control strategies.

There are a variety of lateral controllers, such as geometric and kinematic controller, dynamic controller, optimal controller, model-based controller, and intelligent controller [1]. Geometric and kinematic controllers, such as pure pursuit [12], are developed based on the geometric model with Ackermann steering configuration. This kind of controller is very popular in industry due to its stability and low online computation, but its parameters may suffer from over-tuning [1], and such a controller takes no consideration of vehicle dynamic forces. The dynamic properties of a vehicle can be handled by a dynamic controller [13], in which both wheel slip and wheel cornering are considered. The optimal controller, especially the linear quadratic regulator (LQR) with discrete linear model, is widely reported [14], where the feedback gain is determined by linear quadratic optimization, and it can be computed offline without need of high-cost hardware. In order to estimate the ideal control inputs in various vehicle conditions, model-based controllers are developed [1]. Model predictive control (MPC) is a well-known algorithm, which usually adopts a plant model to predict the response of the vehicle and then chooses the best control input [15]. Due to its complex algorithms, high performance computers are needed. Adaptive and other kinds of intelligent controllers are also investigated [16].

Most of the above control strategies do not take into account the time delays induced by sensors, which has a large impact on the quality and stability of lateral control. Vision-based sensors, such as monocular cameras, are widely used in lane detecting or vehicle localization due to their low cost, and the vehicle-lane information can be obtained reliably through visual algorithms [17,18,19,20,21,22]. However, the computational cost of the visual algorithm is relatively large. Until now, the computing performance of most of the commercialized sensor chips cannot process real-time road information, and uneven time delays occur frequently, where the delays are correlated with the complexity of the scenarios [23,24,25].

For uneven sensor time delays, a feasible method is to increase the update rate of lane information using multi-sensor information fusion and multi-rate Kalman filtering (KF), thus reducing the influence of time delays on the vehicle lateral control [23,24,25,26,27,28,29]. Wang proposed a series of methods for vision-based lateral state estimation of autonomous vehicles considering multi-rate and uneven measurement delay issues, and his proposed algorithm could reduce the root-mean-square error (RMSE) of the vehicle states, such as yaw rate and velocity [23,24,25]. Chung’s team has done valuable work in vehicle state estimation and control method of the scenarios with vision-induced delays [27,28,29,30]. Robust multi-rate lane keeping control scheme is proposed, and multi-rate KF has been developed to estimate vehicle states at a fast rate, in order to resolve the problems caused by slow lane detection [27]. According to experiments, such a control scheme can effectively improve vehicle’s lane keeping performance and reduce the ripple in the yaw rate. In this method, the signal of the inertial measurement unit (IMU) is used in the updating of the lane information. Consider that the signal noise of the IMU is large [31], a high-quality filter is needed. On the other hand, the real-time computing is increased and may enhance the requirement of hardware [32].

In order to balance the robustness under uneven time-delay disturbances and the computational cost, a robust controller is investigated [33,34,35,36,37,38]. There are some successful applications of robust control in vehicle stability control. Shuai [33] and Zhu [34] designed active steering controllers for a four-wheel drive (4WD) vehicle for the random time delay in the network and the effectiveness of the proposed controllers are verified by simulation. Jing and Wang [35] applied a robust output-feedback controller for the vehicle lateral motion control to deal with network-induced delay, and it also considered the effects of tire force saturation. However, insufficient studies have been conducted in robust control of autonomous path tracking under uneven time-delay. In this study, a robust control scheme is designed to compensate sensor-induced uncertainties and improve control performance.

In this paper, a hierarchical vision-based lateral control scheme is proposed for lateral tracking control of autonomous vehicle, where the upper controller is an H_∞_-based LQR controller and the lower controller is based on the logic threshold method. The purpose of the controller is to reduce the disturbance induced by the uneven time delays of visual sensors and give the control of the steering wheel angle strong convergence. On the other hand, the online computing load is kept on a low level, which guarantees its real-time performance. The remaining part of this paper is organized as follows: In Section 2, the vehicle lateral control problem with uneven time delays is introduced and formulated mathematically, and the polytopes of matrices are used to express the nonlinear uncertainties due to time delays. In Section 3, an H_∞_-based LQR controller is presented and solved by linear matrix inequality (LMI) approach. In Section 4, the proposed method is verified by a HIL bench, whereas Section 5 draws the conclusion.

## 2. Problem Formulation

### 2.1. Lane and Vehicle Trajectory Modeling

**Figure 1:**
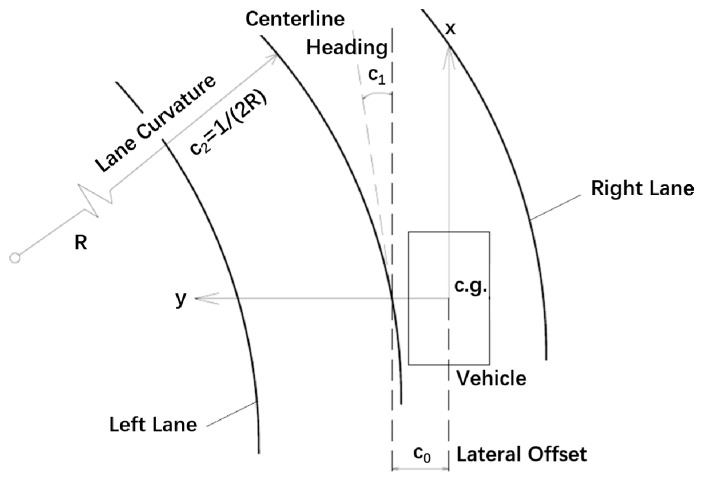
Positional relationship between vehicles and lanes.

In camera-based lateral motion control systems, the camera detects vehicle-lane information in the vehicle’s coordinate system {x,y}, as shown in Figure 1. The parameters detected by the camera include the lane curvature $c_{2}$, the heading angle $c_{1}$ (the orientation error of the vehicle with respect to the road), and the lateral offset $c_{0}$ (the distance of the vehicle’s c.g. from the centerline of the lane) [39]. The lane centerline y(x) can be expressed with polynomial form

(1) $$y\left(x\right)=c_{2}x^{2}+c_{1}x+c_{0}.$$

From the geometric relations [39], we have

(2) $$c_{2}=\frac{1}{2R},\;c_{1}=\psi \;,c_{0}=y\left(0\right)$$

where R is the radius of the lane. The lane polynomial parameter remains unchanged until the next frame of the signal arrives. Assuming that the vehicle is running at constant velocity $V_{x}$ and constant yaw rate $\dot{\psi }$, the vehicle trajectory $f_{v}\left(x\right)$ can be obtained by

(3) $$f_{v}\left(x\right)=\frac{\rho_{v}}{2}x^{2}=\frac{\dot{\psi }}{2V_{x}}x^{2}$$

where x is the longitudinal distance, and $\rho_{v}$ is the curvature of the vehicle trajectory.

### 2.2. Vehicle Modeling for Tracking Control

From Equation (3), the expected lateral displacement of the vehicle $e_{yL_{d}}$ at a preview distance L, and the desired yaw rate ${\dot{\psi }}_{d}$ can be derived as

(4) $$e_{yL_{d}}=\frac{\rho_{v}}{2}L^{2}=\frac{\dot{\psi }}{2V_{x}}L^{2}$$

(5) $${\dot{\psi }}_{d}=\frac{V_{x}}{R}=2c_{2}V_{x}$$

where the vehicle is considered as traveling on a road of constant radius R. Then the desired acceleration can be written as

(6) $$\frac{V_{x}^{2}}{R}=V_{x}{\dot{\psi }}_{d}.$$

The lateral error $e_{y}$ and the yaw error $e_{\psi }$ [40] at the current position of the vehicle are defined by

(7) $${\ddot{e}}_{y}=\left(\ddot{y}+V_{x}\dot{\psi }\right)-\frac{V_{x}^{2}}{R}=\ddot{y}+V_{x}\left(\dot{\psi }-{\dot{\psi }}_{d}\right)$$

(8) $$e_{\psi }=\psi -\psi_{d}$$

By integrating Equation (7), we have

(9) $$\begin{array}{l} {\dot{e}}_{y}=\dot{y}+V_{x}\left(\psi -\psi_{d}\right)\\ e_{y}=y\left(0\right). \end{array}$$

In path tracking, the trajectory of the vehicle may oscillate if only the lateral offset under the current vehicle position is used as control feedback [39]. To solve this problem, the point in front of the vehicle at a certain preview distance L is regarded as the preview point, and then the problem is transformed into the tracking control with preview distance. Considering road geometry, lateral error of the vehicle at the preview point $e_{yL}$ (assuming that the vehicle is running without any yaw rate) can be defined as

(10) $$e_{yL}=e_{y}+L\left(\psi +2c_{2}L\right)=2c_{2}L^{2}+c_{1}L+c_{0}.$$

In fact, taking into account of the preview distance is somewhat similar to the driver’s steering control strategy, considering that driver usually prefers to control the lateral position error at a point (or within a certain distance) in front of the vehicle rather than focusing on the current position [41]. A larger preview distance is usually better than a shorter one, but it is limited by the camera’s detection capability [28]. One feasible way is to set a fixed preview time T so that the preview distance L can be determined by $L=V_{x}T$, which is automatically adjusted according to vehicle speed. At higher speeds, a larger preview distance is used to ensure stability; and at lower speeds, a smaller preview distance is used to suppress the tracking error. After many tests, the preview time was selected as 0.7 s, which could achieve a balance between the vehicle’s driving stability and the tracking error. In fact, in this paper the longitudinal velocity is fixed as 70 km/h during the test, thus the preview distance L is here fixed at about 14 m.

A bicycle model with 2-DOF is used for the lateral dynamics, assuming that a longitudinal controller is working so that the longitudinal velocity keeps constant for a finite period of time. Define the vehicle states as tracking error variables

(11) $$x={\left[\begin{array}{ccccc} e_{yL_{eI}} & e_{yL_{e}} & {\dot{e}}_{y} & e_{\psi } & {\dot{e}}_{\psi } \end{array}\right]}^{T}$$

where $e_{yL_{e}}=e_{yL}-e_{yL_{d}}$, represents the lateral error of the vehicle at the preview point [27], which includes the vehicle yaw rate and the geometry of the road; and $e_{yL_{eI}}=\int_{0}^{t}e_{yL_{e}}d\tau$, represents the integration of the lateral error at the preview point. Neglecting the road bank, and the state space model in tracking error variables x is derived as [27]

(12) $$\begin{array}{l} \dot{x}=\underset{A}{\underset{︸}{\left[\begin{array}{ccccc} 0 & 1 & 0 & 0 & 0\\ 0 & 0 & 1 & 0 & L\\ 0 & 0 & A_{33} & A_{34} & A_{35}\\ 0 & 0 & 0 & 0 & 1\\ 0 & 0 & A_{53} & A_{54} & A_{55} \end{array}\right]}}x+\underset{B}{\underset{︸}{\left[\begin{array}{c} 0\\ 0\\ B_{31}\\ 0\\ B_{51} \end{array}\right]}}u+\underset{B_{w}}{\underset{︸}{\left[\begin{array}{cc} 0 & 0\\ -1 & L\\ 0 & -V_{x}+A_{35}\\ 0 & 0\\ 0 & A_{55} \end{array}\right]}}\underset{w}{\underset{︸}{\left[\begin{array}{c} {\dot{e}}_{yL_{d}}\\ {\dot{\psi }}_{d} \end{array}\right]}}\\ y=\underset{C}{\underset{︸}{\left[\begin{array}{ccccc} 1 & 0 & 0 & 0 & 0\\ 0 & 1 & 0 & 0 & 0\\ 0 & 0 & 0 & 1 & 0\\ 0 & 0 & 0 & 0 & 1 \end{array}\right]}}x \end{array}$$

where

$$\begin{array}{l} A_{33}=-\frac{2C_{\alpha f}+2C_{\alpha r}}{mV_{x}},\;A_{34}=\frac{2C_{\alpha f}+2C_{\alpha r}}{m},A_{35}=-\frac{2C_{\alpha f}l_{f}-2C_{\alpha r}l_{r}}{mV_{x}},\;A_{53}=-\frac{2C_{\alpha f}l_{f}-2C_{\alpha r}l_{r}}{I_{z}V_{x}},\\ A_{54}=\frac{2C_{\alpha f}l_{f}-2C_{\alpha r}l_{r}}{I_{z}},\;A_{55}=-\frac{2C_{\alpha f}l_{f}^{2}+2C_{\alpha r}l_{r}^{2}}{I_{z}V_{x}},B_{31}=\frac{2C_{\alpha f}}{mV_{x}},\;B_{51}=\frac{2C_{\alpha f}l_{f}}{I_{z}}. \end{array}$$

In Equation (12), $C_{\alpha f}(C_{\alpha r})$ is the cornering stiffness of front (rear) tires, m the vehicle mass, $l_{f}(l_{r})$ the distance from the vehicle c.g. to front (rear) axis, $I_{z}$ the vehicle’s yaw moment of inertia. The tracking control can be regarded as a problem of stabilizing the dynamics given by Equation (12), in order to achieve

(13) $$\underset{t\to \infty }{\lim}e_{yL_{e}}=0,\;\underset{t\to \infty }{\lim}{\dot{e}}_{y}=0,\;\underset{t\to \infty }{\lim}e_{\psi }=0,\;\underset{t\to \infty }{\lim}{\dot{e}}_{\psi }=0.$$

Note that **w** is a disturbance vector which disturbs the tracking control [27], especially on a curved lane. Thus, the integrator $e_{yL_{e}}$ is applied to suppress the steady-state error caused by **w**. The input vector **u** is the steering angle of the front wheel $\theta_{f}$.

Taking into account that the actual vehicle controller works at a fixed control cycle, the state-space equation needs to be converted to a discrete form [34], which can be written as

(14) $$\begin{array}{l} x_{k+1}=A_{d}x_{k}+B_{d}u_{k}+B_{wd}w_{k},\\ A_{d}=e^{AT_{s}},\;B_{d}=\int_{0}^{T_{s}}e^{A\left(T_{s}-\tau \right)}d\tau \cdot B,\;B_{wd}=\int_{0}^{T}e^{A\left(T_{s}-\tau \right)}d\tau \cdot B_{w} \end{array}$$

where $x_{k}$ is the state vector, $u_{k}$ is the control input, $w_{k}$ is the disturbance at time $kT_{s}$, and $T_{s}$ is the sample period.

### 2.3. Control Model with Time-Varying Delays

**Figure 2:**
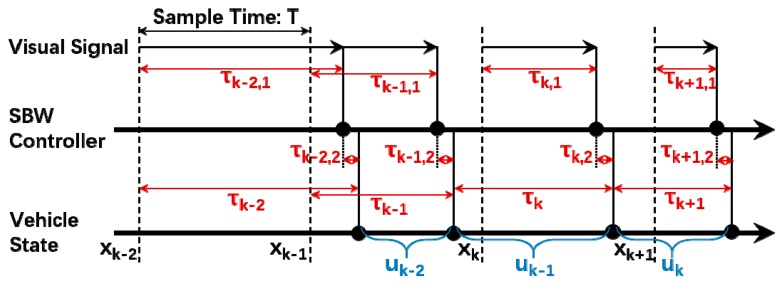
Uneven time delays in the control system, where $\tau_{k_{j},1}$ is induced by visual sensor, and $\tau_{k_{j},2}$ is induced by CAN bus and the actuator response.

In an ideal situation, the states of the system can be obtained in real time. However, there are always time delays in the actual system. The signals of vision sensor are usually transmitted via the controller area network (CAN) on a vehicle, and the time delays induced can be measured by the time stamp of the CAN messages. Furthermore, for the control system, the delays are not only caused by the computing unit of the visual sensors. In fact, there are also random delays in the signal transmission of the CAN [33], and actuators such as the SBW system have response delays [37]. The uneven time delays are shown in Figure 2.

The sum of these delays can be denoted as

(15) $$\tau_{k}=\sum_{i=1}^{n}\tau_{k,i}$$

where $\tau_{k}$ is the total time delay at sample period k, and $\tau_{k,i}$ is the ith component of time delay (such as sensor-induced delay, network-induced delay). In this paper, the total time delays are considered in the following controller. According to the experimental results, it is reasonable to assume the time delays are bounded. Then, the total time delay $\tau_{k}$ can be assumed be distributed in a bounded interval, which can be expressed as

(16) $$0\leq \tau_{k}\leq \tau_{upper}$$

where $\tau_{upper}$ represents the upper bound of the time delay, and it can be written as

(17) $$\tau_{upper}=\left(\lambda +\xi \right)T_{s}$$

where $\lambda \in {\mathbb{Z}}_{+}$ and $\xi \in {\mathbb{R}}_{\left[0,1\right)}$.

Based on the above assumptions, it can be seen that at the time $kT_{s}$, the input $u_{k},u_{k-1},\cdots ,u_{k-\lambda -1}$ may have influence on the system based on the length of time delay, which means that in each control cycle, the system may be affected by the several previous control commands. Therefore, the control model can be transformed into

(18) $$x_{k+1}=A_{d}x_{k}+B_{d}u_{k}+B_{wd}w_{k}+\Delta_{0,k}\left(u_{k-1}-u_{k}\right)+\Delta_{1,k}\left(u_{k-2}-u_{k-1}\right)+\cdots +\Delta_{\lambda ,k}\left(u_{k-\lambda -1}-u_{k-\lambda }\right)$$

where

(19) $$\Delta_{i,k}=\left\{ \begin{array}{ll} 0, & \tau_{k-i}-iT_{s}\leq 0\\ \int_{0}^{\tau_{k-i}-iT_{s}}e^{A\left(T_{s}-\tau \right)}d\tau \cdot B, & 0\leq \tau_{k-i}-iT_{s}\leq T_{s}\\ \int_{0}^{T_{s}}e^{A\left(T_{s}-\tau \right)}d\tau \cdot B, & T_{s}\leq \tau_{k-i}-iT_{s} \end{array}\right..$$

The above uncertainties in Equation (19) can be expressed as a general integral form, where

(20) $$\Gamma \left(x\right)=\int_{0}^{x}e^{A\left(T_{s}-\tau \right)}d\tau .$$

In order to express the system with uncertainties more concisely, the state vector $x_{k}$ can be augmented as $\zeta_{k}$, where

(21) $$\zeta_{k}={\left[\begin{array}{cccc} x_{k}^{T} & u_{k-1}^{T} & \cdots & u_{k-\lambda -1}^{T} \end{array}\right]}^{T}$$

then the system can be expressed in an augmented form

(22) $$\zeta_{k+1}=A_{d,aug}\zeta_{k}+B_{d,aug}u_{k}+B_{wd,aug}w_{k}$$

where

$$\begin{array}{l} A_{d,aug}=\left[\begin{array}{ccccc} A_{d} & \Delta_{0,k}-\Delta_{1,k} & \cdots & \Delta_{\lambda -1,k}-\Delta_{\lambda ,k} & \Delta_{\lambda ,k}\\ 0 & 0 & \cdots & 0 & 0\\ 0 & I & \cdots & 0 & 0\\ \vdots & \vdots & \ddots & \vdots & \vdots \\ 0 & 0 & \cdots & I & 0 \end{array}\right],\\ B_{d,aug}={\left[\begin{array}{cccccc} B_{d}-\Delta_{0,k} & I & 0 & \cdots & 0 & 0 \end{array}\right]}^{T},\\ B_{wd,aug}={\left[\begin{array}{cccccc} B_{wd} & I & 0 & \cdots & 0 & 0 \end{array}\right]}^{T}. \end{array}$$

Until now, the control problem has been described by an augmented discrete model under uneven time delays. Then, the system uncertainties need to be mathematically described.

### 2.4. Description of Time-Delay Uncertainties

In the control design of complex systems, simplification of dynamic characteristics and unknown changes of the parameters will induce uncertainties of the system model. In the system modeling, the system is usually divided into two parts: (1) the nominal system model that ignores the uncertainties; and (2) the uncertainties which are used to describe the uncertain factors. The nominal system model is usually required to be as concise as possible so long it can describe system characteristics; and the uncertainties can be allowed to be complex enough to accommodate as many uncertain factors as possible.

There are mainly two types of model uncertainties: (1) dynamic uncertainty, such as changes in dynamic behavior due to the failure to consider time-varying characteristics and nonlinearity in the input; (2) parameter uncertainties, that is, some physical parameters that are difficult to describe accurately, or the parameters themselves are variable. For the above two types of model uncertainties, there are two methods for describing uncertain systems [33]: Method 1, the matrix polytope model; and Method 2, the affine parameter dependence model. The main difference between them is that, in Method 1, the matrix polytope model is a weighting of the system matrix, where the matrix usually corresponds to uncertain states and has particular physical meaning, but the weighting coefficient often does not have physical meaning. In Method 2, the coefficients in the affine parameter dependence model usually have physical meanings, which are the uncertain parameters of the system. The matrix multiplied with the coefficients usually has no physical meaning. In this paper, the vehicle model is built without considering of time-varying characteristics, which satisfies the applicable scenarios of Method 1 [42]. Therefore, in this study, Method 1 is adopted to describe model uncertainties.

According to the abovementioned description of uncertainties in system with time-varying delays, there is a nonlinear relationship between uncertainties and time delay. Therefore, it is impossible to apply the polytopic inclusion directly. Linearization is a feasible way to describe the uncertainties in a linear form [43], and the Taylor series expansion is used here. In this way, the Equation (20) can be expanded as

(23) $$\Gamma \left(x\right)=\Gamma \left(0\right)+\dot{\Gamma }\left(0\right)x+\ddot{\Gamma }\left(0\right)\frac{x^{2}}{2!}+\cdots +\frac{d^{q}\Gamma }{dx^{q}}\left(0\right)\frac{x^{q}}{q!}+\cdots =-\sum_{q=1}^{\infty }\frac{{\left(-x\right)}^{q}}{q!}A^{q-1}e^{AT_{s}}.$$

With the first h terms, Γ(x) can be expressed as

(24) $$\Gamma \left(x\right)=-\sum_{q=1}^{h}\frac{{\left(-x\right)}^{q}}{q!}A^{q-1}e^{AT_{s}}+\Theta^{h}$$

where $\Theta^{h}$ is a high-order remainder

(25) $$\Theta^{h}=-\sum_{q=h+1}^{\infty }\frac{{\left(-x\right)}^{q}}{q!}A^{q-1}e^{AT_{s}}.$$

With a proper h, the remainder $\Theta^{h}$ can be neglected, and the h-order approximation of the uncertainties are approximated as a polynomial

(26) $$\Gamma^{h}\left(x\right)=-\sum_{q=1}^{h}\frac{{\left(-x\right)}^{q}}{q!}A^{q-1}e^{AT_{s}}.$$

For the convenience of the subsequent mathematical description of the uncertainties, the following notations are defined as

(27) $$G_{q}=\frac{{\left(-1\right)}^{q+1}}{q!}A^{q-1}e^{AT_{s}}$$

and

(28) $$\left\{ \begin{array}{l} \phi_{j,1}={\left[\begin{array}{ccccc} {\underline{\rho }}^{h}I & {\underline{\rho }}^{h-1}I & \cdots & {\underline{\rho }}^{2}I & \underline{\rho }I \end{array}\right]}^{T}\\ \phi_{j,2}={\left[\begin{array}{ccccc} {\underline{\rho }}^{h}I & {\underline{\rho }}^{h-1}I & \cdots & {\underline{\rho }}^{2}I & {\bar{\rho }}_{j}I \end{array}\right]}^{T}\\ \phi_{j,3}={\left[\begin{array}{ccccc} {\underline{\rho }}^{h}I & {\underline{\rho }}^{h-1}I & \cdots & {\bar{\rho }}_{j}^{2}I & {\bar{\rho }}_{j}I \end{array}\right]}^{T}\\ \vdots \\ \phi_{j,h}={\left[\begin{array}{ccccc} {\underline{\rho }}^{h}I & {\bar{\rho }}_{j}^{h-1}I & \cdots & {\bar{\rho }}_{j}^{2}I & {\bar{\rho }}_{j}I \end{array}\right]}^{T}\\ \phi_{j,h+1}={\left[\begin{array}{ccccc} {\bar{\rho }}_{j}^{h}I & {\bar{\rho }}_{j}^{h-1}I & \cdots & {\bar{\rho }}_{j}^{2}I & \bar{\rho }I \end{array}\right]}^{T} \end{array}\right.$$

where q=1,2,⋯h; j=0,1; $\underline{\rho }=0$; ${\bar{\rho }}_{0}=T_{s}$; and ${\bar{\rho }}_{1}=\xi T_{s}$, and then the uncertain terms $\Delta_{i,k}$ can be included into a polytope with vertices [42]. With the notations defined in Equation (27) and Equation (28), the vertices of the convex polytope can be expressed as

(29) $$\left\{ \begin{array}{l} {\bar{\Delta }}_{0.i}=\left[\begin{array}{ccccc} G_{h} & G_{h-1} & \cdots & G_{2} & G_{1} \end{array}\right]\phi_{0,i}B\\ {\bar{\Delta }}_{1.i}=\left[\begin{array}{ccccc} G_{h} & G_{h-1} & \cdots & G_{2} & G_{1} \end{array}\right]\phi_{1,i}B \end{array}\right.$$

where ${\bar{\Delta }}_{0.i}$ and ${\bar{\Delta }}_{1.i}$ can be regarded as a group of linear bases. Then, the uncertainties $\Delta_{i,k}$ can be expressed by the linear combination of the vertices

(30) $$\begin{array}{l} \left\{ \begin{array}{l} \Delta_{i.k}=\sum_{l=1}^{h+1}\sigma_{i,l}\left(k\right){\bar{\Delta }}_{0,l},\;i=0,1,\cdots ,\lambda -1\\ \Delta_{i.k}=\sum_{l=1}^{h+1}\sigma_{i,l}\left(k\right){\bar{\Delta }}_{1,l},\;i=\lambda \end{array}\right.\\ \left\{ \begin{array}{l} \sum_{l=1}^{h+1}\sigma_{i,l}\left(k\right)=1\\ \sigma_{i,l}\left(k\right)>0 \end{array}\right.,\forall l=1,2,\cdots ,h+1,\forall k\in {\mathbb{Z}}^{+} \end{array}$$

where $\sigma_{i,l}\left(k\right)$ is the time-varying coefficient, without any physics meaning. Note that for the h-order approximation of the uncertainties, the convex polytope has ${\left(h+1\right)}^{\lambda +1}$ vertices

(31) $$\left\{A_{d,aug,i},B_{d,aug,i},B_{wd,aug,i}\right\}\left(i=1,2,\cdots ,{\left(h+1\right)}^{\lambda +1}\right)$$

where

$$\begin{array}{l} A_{d,aug,i}=\left[\begin{array}{ccccc} A_{d} & \Delta_{0,l_{0}}-\Delta_{1,l_{1}} & \cdots & \Delta_{0,l_{\lambda -1}}-\Delta_{1,l_{\lambda }} & \Delta_{1,l_{\lambda }}\\ 0 & 0 & \cdots & 0 & 0\\ 0 & I & \cdots & 0 & 0\\ \vdots & \vdots & \ddots & \vdots & \vdots \\ 0 & 0 & \cdots & I & 0 \end{array}\right],\\ B_{d,aug,i}={\left[\begin{array}{cccccc} B_{d}-\Delta_{0,l_{0}} & I & 0 & \cdots & 0 & 0 \end{array}\right]}^{T},\\ \forall l_{0}=1,2,\cdots ,h+1,\forall l_{1}=1,2,\cdots ,h+1. \end{array}$$

Now the linear description of uncertainties has been obtained, and control method will be given in Section 3.

## 3. Control Synthesis

**Figure 3:**
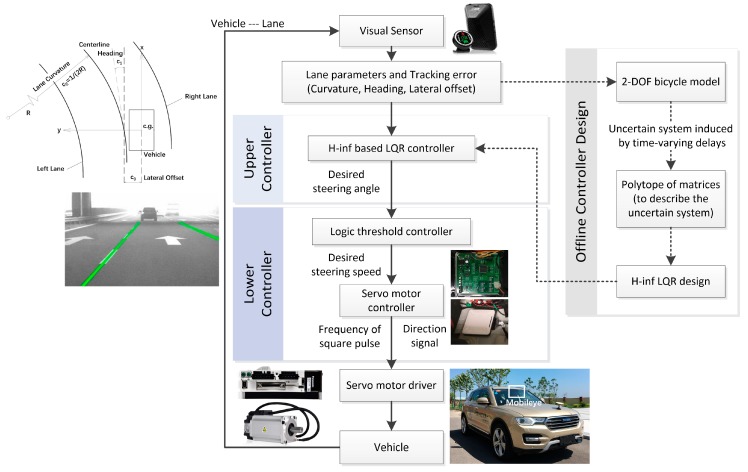
Control scheme of the lateral tracking task.

As shown in Figure 3, the lateral tracking control of autonomous vehicle scheme is handled by a hierarchical structure, which includes: (1) an upper controller to determine the desired steering wheel angle based on H_∞_-based LQR control method, where the delay uncertainties are described by polytopic and the feedback series are solved in the form of LMIs; and (2) a lower controller, where the logic threshold algorithm is adopted for the closed-loop steering angle control, and the control output is transmitted to the servo motor.

### 3.1. Upper Controller

The upper controller is designed to calculate the desired front wheel angle (or desired steering wheel angle) based on the vehicle status and road parameters. In this paper, an H_∞_-based LQR controller is developed for the lateral tracking task under the uneven time delays. The input of the upper controller is the tracking error, while the output is the desired steering wheel angle $\theta_{sw,des}\left(k\right)$.

#### 3.1.1. H_∞_-Based LQR Control 

The purpose of the lateral control is to minimize both the tracking error and the control input. A performance index J is adopted in quadratic form, which is expressed as

(32) $$J=\sum_{k=0}^{\infty }\left(e_{k}^{T}Qe_{k}+u_{k}^{T}Ru_{k}\right)$$

where $e_{k}$ is the tracking error at sample time k, and Q, R are weighted matrices. Furthermore, the index can be transformed into the 2-norm of the $z_{k}$, where

(33) $$\begin{array}{l} z_{k}=Q_{aug}\zeta_{k}+R_{aug}u_{k},\\ Q_{aug}=\left[\begin{array}{cccc} Q^{1/2} & 0 & \cdots & 0\\ 0 & 0 & \cdots & 0 \end{array}\right],\;R_{aug}=\left[\begin{array}{c} 0\\ R^{1/2} \end{array}\right]. \end{array}\;$$

Since the disturbance w is bounded in $l_{2}$ space, an $H_{\infty }$ performance index η is introduced as [33]

(34) $${\left\|z\right\|}_{2}^{2}<\eta^{2}{\left\|w\right\|}_{2}^{2}.\;$$

Then the optimization control problem is converted into the design of the $H_{\infty }$ controller for the following system

(35) $$\begin{array}{l} \zeta_{k+1}=A_{d,aug}\zeta_{k}+B_{d,aug}u_{k}+B_{wd,aug}w_{k}\\ z_{k}=Q_{aug}\zeta_{k}+R_{aug}u_{k}. \end{array}\;$$

The objective is to get the state-feedback gains, which can make the closed-loop system asymptotically stable and minimize η while it satisfies Equation (35). In order to solve the minimization problem, the following *Lemma* is introduced.

Lemma [44]: For the designed controller, the closed-loop system is stable with a given η if there exist positive definite matrices $P=P^{T}>0$, and M which satisfies

(36) $$\left[\begin{array}{cccc} -P & 0 & \left(A_{d,aug}+B_{d,aug}K\right)M & B_{wd,aug}\\ {}* & -I & \left(Q_{aug}+R_{aug}K\right)M & 0\\ {}* & * & P-M-M^{T} & 0\\ {}* & * & * & -\eta^{2}I \end{array}\right]<0.$$

#### 3.1.2. Vehicle Lateral Controller

Based on the above Lemma, the vehicle lateral controller can be obtained. Consider that each vertex corresponds to a system, ${\left(h+1\right)}^{\lambda +1}$ systems can be obtained

(37) $$\zeta_{k+1}=A_{d,aug,i}\zeta_{k}+B_{d,aug,i}u_{k}+B_{wd,aug,i}w_{k}\;$$

where $i=1,2,\cdots ,{\left(h+1\right)}^{\lambda +1}$. The purpose is to solve an feedback gain K, which satisfies the conditions

(38) $$\begin{array}{l} \left[\begin{array}{cccc} -P & 0 & \left(A_{d,aug,i}+B_{d,aug,i}K\right)M & B_{wd,aug}\\ {}* & -I & \left(Q_{aug}+R_{aug}K\right)M & 0\\ {}* & * & P-M-M^{T} & 0\\ {}* & * & * & -\eta^{2}I \end{array}\right]<0\\ \forall i=1,2,\cdots ,{\left(h+1\right)}^{\lambda +1}. \end{array}\;$$

For the sake of simplicity, let Y=KM, and the design of the controller can be transformed as

(39) $$\begin{array}{l} \underset{P,M,Y,\eta }{\min}\eta^{2}\\ s.t.\left[\begin{array}{cccc} -P & 0 & A_{d,aug,i}M+B_{d,aug,i}Y & B_{wd,aug}\\ {}* & -I & Q_{aug}M+R_{aug}Y & 0\\ {}* & * & P-M-M^{T} & 0\\ {}* & * & * & -\eta^{2}I \end{array}\right]<0\\ \forall i=1,2,\cdots ,{\left(h+1\right)}^{\lambda +1} \end{array}\;$$

where a smaller η accompanies a smaller z, which also means that the lateral tracking error is smaller. Equation (39) is a minimization of a linear objective function with constraints of LMIs. Such problem can be solved offline by MATLAB LMI Toolbox, and the state feedback matrix can be derived by $K=YM^{-1}$. Note that K is a constant matrix, which indicates that it can be solved offline without increasing the computing load of the hardware. Then the desired steering angle of the front wheel at sample time k can be obtained as $\theta_{f,des}\left(k\right)$, where

(40) $$\theta_{f,des}\left(k\right)=K\zeta_{k}\;$$

Assuming that the steering wheel angle is in linear relationship with the front wheel angle, the desired steering wheel angle $\theta_{sw,des}\left(k\right)$ can be obtained

(41) $$\theta_{sw,des}\left(k\right)=\mu \theta_{f,des}\left(k\right)\;$$

where μ is the ratio of the steering wheel angle to the front wheel angle, and in this paper it is 17.4.

### 3.2. Lower Controller

The lower controller is used to calculate the desired speed of rotation of the steering motor based on the desired front wheel rotation angle (or desired steering wheel angle) given by upper controller. In this paper, a logic threshold-based controller is developed, which has strong convergence and good robustness under different operating conditions. The algorithm framework is simple, easy to maintain, and it is convenient for parameter calibration in real vehicles. Here, the input of the lower controller is the desired wheel steering angle, and the output is the frequency of the square wave (to control the motor speed) and the direction signal (to control the motor direction).

The angle difference Δθ between the target steering wheel angle $\theta_{sw,des}\left(k\right)\theta_{tar}$ and the real steering wheel angle $\theta_{sw,real}\left(k\right)$ is obtained by

(42) $$\Delta \theta \left(k\right)=\theta_{sw,des}\left(k\right)-\theta_{sw,real}\left(k\right)$$

Then the desired speed of the steering wheel can be obtained based on angle difference Δθ(k)

(43) $${\dot{\theta }}_{des}\left(k\right)=\left\{ \begin{array}{ll} \frac{\left|\Delta \theta \left(k\right)\right|}{\lambda_{upper}\times T_{s}}, & \left|\Delta \theta \left(k\right)\right|>\Theta_{upper}\\ \frac{\left|\Delta \theta \left(k\right)\right|}{\lambda_{mid}\times T_{s}}, & \Theta_{upper}\geq \left|\Delta \theta \left(k\right)\right|>\Theta_{mid}\\ \frac{\left|\Delta \theta \left(k\right)\right|}{\lambda_{lower}\times T_{s}}, & \Theta_{mid}\geq \left|\Delta \theta \left(k\right)\right|>\Theta_{lower}\\ \frac{\left|\Delta \theta \left(k\right)\right|}{\lambda_{least}\times T_{s}}, & \left|\Delta \theta \left(k\right)\right|\leq \Theta_{lower} \end{array}\right.$$

where $\Theta_{upper}$, $\Theta_{mid}$, and $\Theta_{lower}$ are the threshold of large angle difference, the threshold of middle angle difference, and the threshold of small angle difference, respectively; $\lambda_{upper}$, $\lambda_{mid}$, $\lambda_{lower}$, and $\lambda_{least}$ are the conversion factors of the motor speed corresponding to each threshold; ${\dot{\theta }}_{des}\left(k\right)$ is the desired steering wheel angle; $T_{s}$ is the sampling period. Note that the value of $\lambda_{upper}$, $\lambda_{mid}$, $\lambda_{lower}$, and $\lambda_{least}$ are successively larger. The purpose is to ensure that the steering motor can approach the desired angle quickly when the angle difference is large, and at the same time ensure that the steering motor can smoothly and steadily approach the desired angle at a lower rotation speed to avoid any overshooting or oscillation due to system inertia and excessive speed.

In actual scenarios, the rotation speed of the steering wheel is limited, and an excessive rotation speed may damage the steering system or make the vehicle unstable; and on the other hand, if the SBW system is frequently involved in the control while the angle difference Δθ(k) is very small, it may cause oscillations in the steering angle. Therefore, the desired speed is limited as ${\bar{\dot{\theta }}}_{des}\left(k\right)$, which can be expressed as

(44) $${\bar{\dot{\theta }}}_{des}\left(k\right)=\left\{ \begin{array}{ll} {\dot{\Theta }}_{upper}, & {\dot{\theta }}_{des}\left(k\right)>{\dot{\Theta }}_{upper}\\ {\dot{\theta }}_{des}\left(k\right), & {\dot{\Theta }}_{upper}\geq {\dot{\theta }}_{des}\left(k\right)\geq {\dot{\Theta }}_{lower}\\ 0, & {\dot{\Theta }}_{lower}>{\dot{\theta }}_{des}\left(k\right)>-{\dot{\Theta }}_{lower}\\ {\dot{\theta }}_{des}\left(k\right), & -{\dot{\Theta }}_{lower}\geq {\dot{\theta }}_{des}\left(k\right)\geq -{\dot{\Theta }}_{upper}\\ -{\dot{\Theta }}_{upper}, & {\dot{\theta }}_{des}\left(k\right)<-{\dot{\Theta }}_{upper} \end{array}\right.$$

where ${\dot{\Theta }}_{upper}$ is the maximum limit of the rotation speed, and ${\dot{\Theta }}_{lower}$ is the minimum rotation speed. Furthermore, the frequency of the square wave $f_{output}\left(k\right)$ is calculated by ${\bar{\dot{\theta }}}_{des}\left(k\right)$

(45) $$f_{output}\left(k\right)=\frac{{\bar{\dot{\theta }}}_{des}\left(k\right)C_{mot}i_{mot}}{360{}^{\circ}}\times F_{calb}$$

where $C_{mot}$ is the factor of the motor rotation, and in this paper it is set to 10,000 (the steering motor rotates 1 revolution while 10,000 pulses are received); $i_{mot}$ is the transmission ratio between the steering motor and the steering wheel, 16; $F_{calb}$ is the frequency coefficient, which is used to correct the frequency error due to the output signal error of the hardware.

In addition, the direction of motor rotation is determined according to the angle difference

(46) $$\delta \left(k\right)=\left\{ \begin{array}{ll} CW, & {\bar{\dot{\theta }}}_{des}\left(k\right)>0\\ CCW, & {\bar{\dot{\theta }}}_{des}\left(k\right)\leq 0 \end{array}\right.$$

where CW represents clockwise rotation and the voltage of the direction signal is 5 V; and CCW represents counter-clockwise rotation, where the voltage of the direction signal is 0 V.

## 4. Experiments and Discussions

### 4.1. Verification of the Lower Controller Based on Vehicle Experiment

The tracking accuracy of the lower controller needs to be verified in order to discuss the proposed control scheme. The experiment was conducted on a vehicle equipped with SBW system. A step desired steering angle and a random desired steering angle were applied to the lower controller to verify its angular accuracy.

#### 4.1.1. Platform and Test Conditions

**Figure 4:**
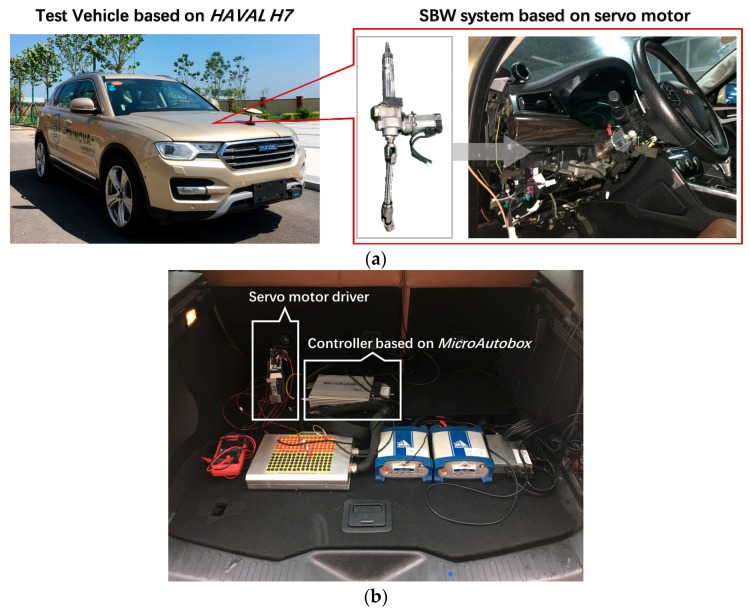
Experiment platform: (**a**) Test vehicle restructured from HAVAL H7, which is equipped with a servo motor-based SBW system; (**b**) The controller based on the dSPACE-MicroAutobox and the servo motor driver, while most of the devices are mounted in the trunk of the test vehicle.

The experiment was conducted on a test vehicle which was restructured from HAVAL H7, and as shown in Figure 4a, a SBW system was designed by adding a group of worm gear and a servo motor on the steering column. The dSPACE-MicroAutobox was used as the lower controller. In fact, any cheap computing units such as microcontrollers could be used as the lower controller due to the small amount of real-time computations. As shown in Figure 4b, the devices such as the controllers and motor driver were mounted in the trunk of the test vehicle.

The calibrated parameters of the lower controller are listed in Table 1, and the experiments with a step desired steering angle and a random desired steering angle were conducted to verify the performance of the lower controller.

#### 4.1.2. Results and Discussions

**Figure 5:**
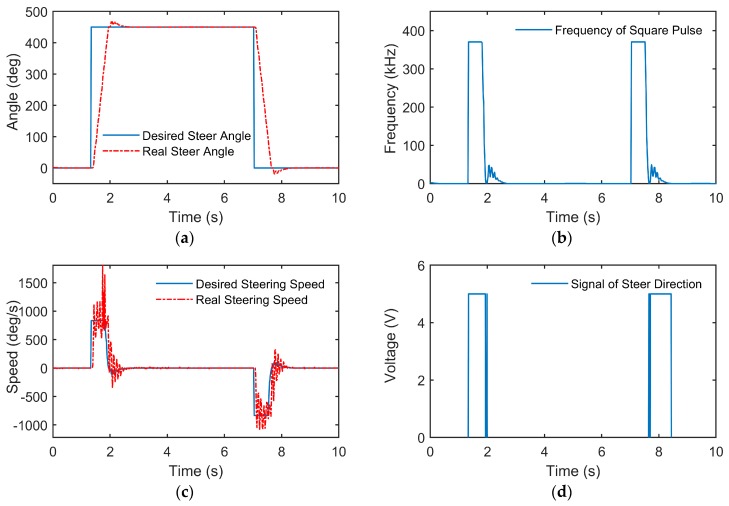
Experiment results of the step desired steering angle: (**a**) real steering wheel angle compared with the desired steering wheel angle; (**b**) frequency of the square pulse output by the lower controller; (**c**) real steering wheel speed compared with the desired steering wheel speed; (**d**) voltage of the signal of steer direction output by the lower controller.

**Figure 6:**
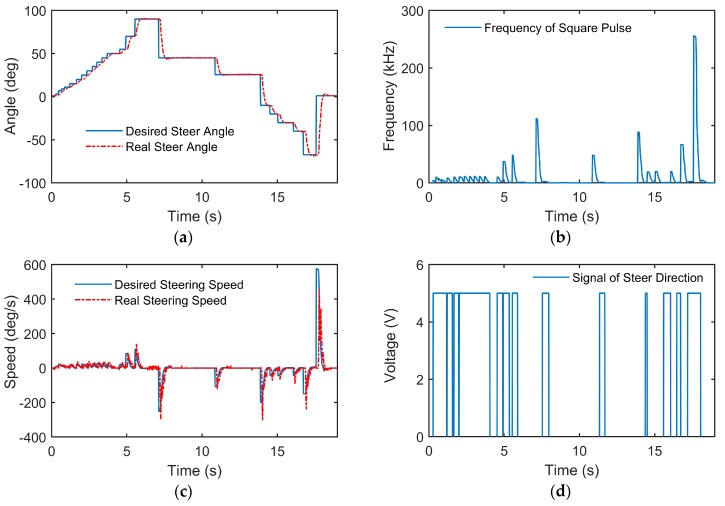
Experiment results of the random desired steering angle: (**a**) real steering wheel angle compared with the desired steering wheel angle; (**b**) frequency of the square pulse output by the lower controller; (**c**) real steering wheel speed compared with the desired steering wheel speed; (**d**) voltage of the signal of steer direction output by the lower controller.

Experiments with a step desired steering angle and a random desired steering angle were conducted, and the results are shown in Figure 5 and Figure 6, respectively.

According to the results of the above two cases of experiments, the lower controller could achieve accurate tracking of the steering wheel angle with small steady state error and small overshooting in dynamic process. Taking the step experiment as an example, the steering motor was set to the highest speed while the desired steering angle turned 450°, with a series of high frequency square wave commands produced by the lower controller (in fact, the frequency of the square pulse is limited to about 833 kHz). After reaching the desired angle, the frequency of the square wave decreased, and the steering angle converged rapidly and stabilized near the desired angle. As seen from Figure 5c, some oscillation occurs in the steering speed around 1.5–2.5 s and 7–8 s. This is mainly because that the steering wheel rotates at a very high speed (higher than 800°/s) in order to achieve the desired angle quickly, and the steering load changes greatly due to nonlinear factors such as the self-aligning torque of the tire. Furthermore, the steering speed is estimated by the differential of the steering angle, which also increases the oscillation of the value of the steering speed. Besides, it can be seen from Figure 5a that the steering angle changes smoothly and evenly, therefore the control performance is quite acceptable.

According to Figure 5b and Figure 6b, the controller almost stops sending control input to the motor when the desired angle remains unchanged for a long period and the real steering angle has reached steady state. This is due to the setting of the dead zone of the steering speed $\left(-{\dot{\Theta }}_{lower},{\dot{\Theta }}_{lower}\right)$, which can decrease the oscillation of the steering motor. The detailed indices of the controller performance are listed in Table 2.

### 4.2. HIL Experiment for the Lateral Control Scheme

The performance of the proposed control scheme for the vehicle lateral control is verified in this subsection. Considering that experiments of path tracking at high speed can be dangerous, a HIL platform was adopted to simulate the experiments, and the proposed H_∞_-based LQR controller was compared with a conventional LQR controller.

#### 4.2.1. Platform

**Figure 7:**
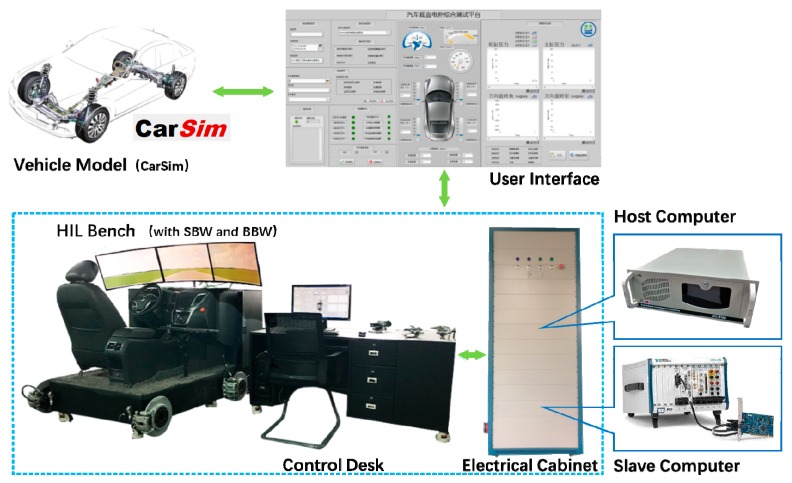
HIL bench with wire-controlled chassis: architecture.

**Figure 8:**
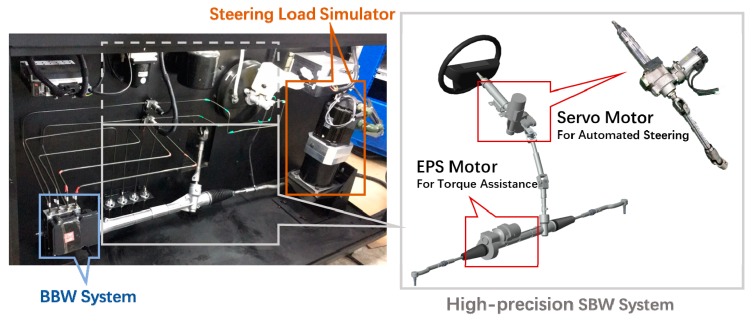
HIL bench with wire-controlled chassis: actuators.

In this paper, a HIL bench with wire-controlled chassis was used for experimental verification of the proposed control algorithms, and the components of the bench is shown in Figure 7. A full-size SUV (suburban utility vehicle) model was adopted provided by CarSim. As shown in Figure 8, the bench is equipped with a SBW system, which is equipped with a high-precision servo steering motor. The processor of the platform is made up of a host computer and a slave computer. The host computer is an IPC (industrial personal computer) which is used to control the HIL bench and display the real-time animation and data, and the user interface was designed by LabVIEW. The slave computer was a PXI (peripheral component interconnect extensions for instrumentation) system by National Instruments. The signal acquisition was conducted by LabVIEW DAQ, and the controller algorithm was implemented by Simulink and LabVIEW MIT. The CarSim real-time vehicle model worked on the slave computer.

#### 4.2.2. Experiment Condition

**Figure 9:**
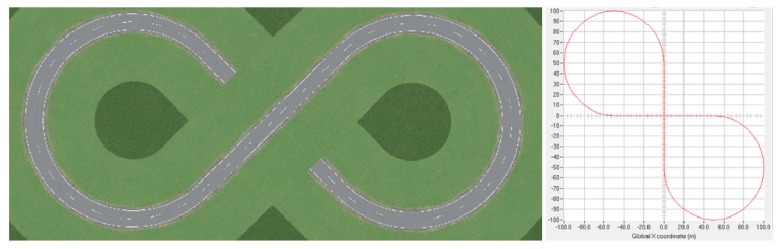
Test road: A cycle lane shaped like figure-eight.

As shown in Figure 9, a cycle lane shaped like a figure-eight is used as test road, while the longitudinal velocity is fixed as 70 km/h, and the sampling time $T_{s}$ is set as 60 ms. The weighted matrices of the proposed controller are $Q=diag\left(\begin{array}{ccccc} 1000, & 2500, & 1, & 100, & 1 \end{array}\right)$ and R=diag(10000). A conventional LQR controller was used for comparison, where the weighted matrices are $Q^{\prime }=diag\left(\begin{array}{ccccc} 60, & 2500, & 1, & 100, & 1 \end{array}\right)$ and $R^{\prime }=diag\left(10000\right)$. (Some modifications were made in the $Q^{\prime }$, because if $Q^{\prime },R^{\prime }$ were made equal to Q,R, the control result of the conventional LQR would not be convergent.)

#### 4.2.3. Results and Discussions

**Figure 10:**
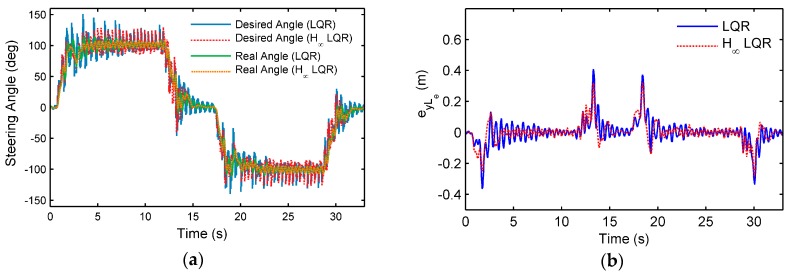

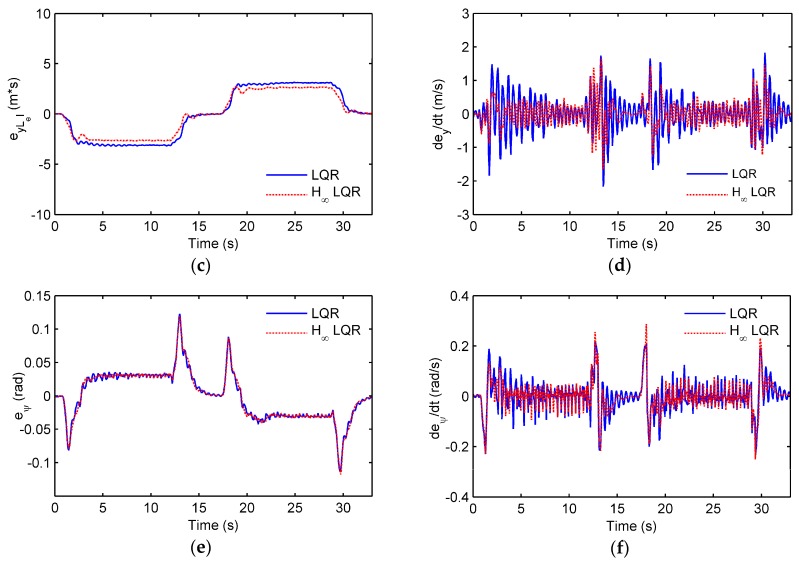
Experiment results of the proposed upper controller, compared with a conventional LQR controller: (**a**) desired steering wheel angle and the real steering wheel angle; (**b**) lateral error at the preview point; (**c**) integration of the lateral error at the preview point; (**d**) differential of the lateral error; (**e**) heading error; (**f**) differential of the heading error.

**Figure 11:**
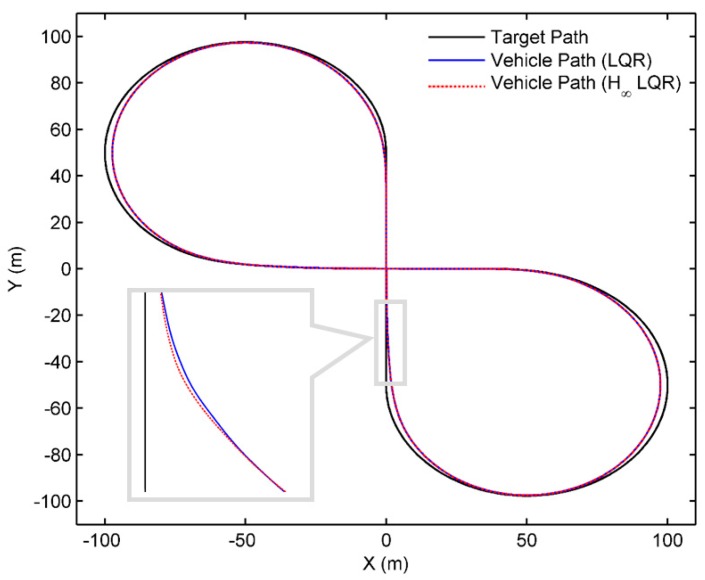
Vehicle path with the designed target lane shaped like figure-eight.

Comparison of the tracking performance between the proposed controller (Case 1) and the conventional LQR controller (Case 2) is given in Figure 10, and the vehicle path is shown in Figure 11. Both controllers can make good tracking to the centerline of the road. The average lateral error at preview point is 0.0427 m for Case 1, and 0.0429 m for Case 2, while the peak value is 0.2962 m and 0.4011 m, respectively. Compared with the conventional LQR controller, the proposed controller has fewer oscillations in the output of the steering wheel angle when the road curvature is large. Vehicle states such as $e_{yL_{e}}$, ${\dot{e}}_{y}$, and ${\dot{e}}_{\psi }$ have fewer oscillations, correspondingly. The performance was evaluated by RSME, and as shown in Table 3, in most of vehicle states the proposed controller provides better performance (while the RMSE of $e_{\psi }$ has a slight increase), which shows the proposed controller’s better robustness and performance. The overall comparison of the proposed controller and the conventional LQR is shown in Table 4, while the comparison with other controllers (such as the kinematic controller, model-based controller, etc.) can be found in [1].

## 5. Conclusions

In this paper, a hierarchical steering control scheme to compensate visual sensor-induced uneven time delays is proposed for the lateral tracking control of the autonomous vehicle. An upper controller is designed with an H_∞_-based LQR algorithm, aiming to determine the steering wheel angle with low cost in online computation, where the nonlinear uncertainties induced by uneven time delays are linearized by Taylor expansion, and the system is augmented to describe the uncertainties with polytopic inclusions. A lower controller with logic threshold method is used for the high-precision tracking of the steering wheel angle. Vehicle experiments were conducted, and the lower controller was proven capable of achieving accurate tracking of the steering wheel angle with small error and overshooting, while the steady-state angular error is less than 0.1° and the overshooting is less than 4.5%. The upper controller was verified with HIL experiments. According to the experiment results, the proposed controller shows better tracking performance compared with the conventional LQR controller, while the average lateral error at preview point is reduced from 0.0429 m to 0.0427 m, and the peak value is reduced from 0.4011 m to 0.2962 m, respectively. Moreover, the proposed controller has fewer oscillations than the conventional LQR in both the controller’s output and the vehicle states, which may improve the ride comfort of the autonomous vehicle. Compared with other kinds of conventional controllers (such as kinematic and dynamic controller, model-based controller), the proposed control scheme well balances the predicted performance with the amount of online calculation, and thus provides a potential low-cost solution for lateral control of autonomous driving.

## Figures and Tables

**Table 1:** Calibrated parameters of the lower controller.

Parameter	Value	Meaning
$\Theta_{upper}$	180°	Threshold of large angle difference
$\Theta_{mid}$	90°	Threshold of mid angle difference
$\Theta_{lower}$	10°	Threshold of small angle difference
$F_{calb}$	1.028	Frequency coefficient
$\lambda_{upper}$	25	Conversion factor of the motor speed
$\lambda_{mid}$	60	Conversion factor of the motor speed
$\lambda_{lower}$	90	Conversion factor of the motor speed
$\lambda_{least}$	110	Conversion factor of the motor speed
${\dot{\Theta }}_{upper}$	830°/s	Maximum limit of the rotation speed
${\dot{\Theta }}_{lower}$	0.1°/s	Minimum limit of the rotation speed

**Table 2:** Control performance of the proposed lower controller in step input case.

Index	Value	Meaning
Overshoot	4.5%	The percentage of the angle value that the real angle exceeds the desired angle during the rotation of the steering wheel.
Steady-state angular error	0.1°	Steady-state error when the real angle of the steering wheel achieves to the desired angle.
Maximum time delay	70 ms	Maximum time difference between the time when the desired angle command is transmitted on the CAN bus and the time when the real angle starts to change.

**Table 3:** Control performance (RMSE) of the proposed controller compared with LQR controller.

Controller	$\mathrm{RMSE}\;\mathrm{of}\;e_{yL_{eI}}$	$\mathrm{RMSE}\;\mathrm{of}\;e_{yL_{e}}$	$\mathrm{RMSE}\;\mathrm{of}\;{\dot{e}}_{y}$	$\mathrm{RMSE}\;\mathrm{of}\;e_{\psi }$	$\mathrm{RMSE}\;\mathrm{of}\;{\dot{e}}_{\psi }\;$
H_∞_-based LQR	1.9598	0.0706	0.4283	0.0374	0.0623
Conventional LQR	2.3415	0.0770	0.5107	0.0370	0.0667
**Optimization**	16.3%	8.3%	16.1%	−1.3%	6.6%

**Table 4:** Comparison between the proposed controller and the conventional LQR controller.

Controller	H_∞_-based LQR	Conventional LQR
Performance	It shows better path-tracking performance, and has smaller position error, less chattering during driving.	The path-tracking can be completed with acceptable position error, but the vehicle chatters a lot, which may reduce ride comfort.
Implementation considerations	Calculations can be implemented offline, with no need for high computational performance of hardware. However, the range of the sensor’s time-delay needs to be known by prior experiments.	Calculations can be implemented offline, and real-time computing load is almost the same as the proposed method in this paper.
Complexity	Mathematical principle is more complicated, but the control law in real-time is simple.	Mathematical principle is simpler, and the control law in real-time is simple.
Stability	Both controllers are stable because they are mathematically proven. However, since the dynamic model of the vehicle is linear, when the vehicle is running under extreme non-linear conditions, the model may have a large error compared with vehicle’s response characteristics in real scenarios and the controller may be unstable.	
Re-usability	The controller is reusable in other plants when the response characteristics of the vehicle and the time-delay characteristics of the vision sensor experience no significant changes.	The controller is reusable when the response characteristics of the vehicle do not change significantly.
